# Distress Response to the Failure to an Insoluble Anagrams Task: Maladaptive Emotion Regulation Strategies in Binge Drinking Students

**DOI:** 10.3389/fpsyg.2017.01795

**Published:** 2017-10-18

**Authors:** Marie Poncin, Nicolas Vermeulen, Philippe de Timary

**Affiliations:** ^1^Psychological Sciences Research Institute, Université catholique de Louvain, Louvain-la-Neuve, Belgium; ^2^Fund for Scientific Research (F.R.S.-F.N.R.S.), Brussels, Belgium; ^3^Department of Adult Psychiatry, Cliniques Universitaires Saint-Luc, Brussels, Belgium; ^4^Institute of Neuroscience, Université catholique de Louvain, Brussels, Belgium

**Keywords:** binge drinking, self-failure, self-regulation, self-consciousness, self-related sensitivity

## Abstract

**Background:** Emotion regulation refers to the attempt to influence the latency, magnitude, and duration of an emotion, and to modify the experiential, behavioral, or physiological components of the emotional response. In situations of personal failure, individuals, and in particular those who present a tendency to self-focus, may experience intense emotional distress. Individuals who lack proper adaptive emotion regulation strategies may engage in activities leading to immediate pleasure, such as alcohol drinking, in order to escape the self-relevance of emotional experiences. This self-awareness theory of drinking has been shown explain relapses in self-focused alcohol-dependent individuals in situations of personal failure, after detoxification. Such relapses support the existence of maladaptive emotion regulation strategies in alcohol dependence. As binge drinking may be considered as an early stage of alcohol-use-disorder, the aim of this study was to explore the relationship between emotional distress, self-regulation and self-consciousness in binge drinkers (BD).

**Methods:** Fifty-five students (32 BD and 23 controls) completed different questionnaires related to the self (self-consciousness and self-regulation questionnaires) and were exposed to a situation of self-failure (insoluble anagrams).

**Results:** The distress induced by the anagrams task was more related to self-blame, ruminations and maladaptive emotion regulation strategies in BD than in controls. Emotional distress was related to less positive refocusing, refocusing on planning, and adaptive emotion regulation strategies among the control group with less public self-consciousness. Emotional distress was related to more positive refocusing, positive reappraisal, refocusing on planning, and adaptive emotion regulation strategies among control participants with higher public self-consciousness. Low self-conscious BD who experienced anagram distress used less acceptance and less refocusing on planning strategies. Conversely, high self-conscious BD used more refocusing on planning strategies when experiencing anagram distress.

**Conclusion:** This study suggests a relationship between emotional distress and self-regulation, in BD only. Moreover, public self-consciousness appears to be a disposition that motivates non-BD to improve actions and attitudes to meet self-standards. Finally, this study suggests a minor role of self-consciousness in the relationship between self-regulation and emotional distress in BD. Finally, low private/public self-consciousness in the binge drinking group may also be related to more maladaptive emotion regulation strategies.

## Introduction

Emotion regulation refers to the attempt to influence the latency, magnitude, and duration of an emotion, and to modify the experiential, behavioral, or physiological components of the emotional response (Gross, 2014). In his process model, Gross (2014) highlights five emotion regulation processes: (1) situation selection, (2) situation modification, (3) attentional deployment, (4) cognitive change, and (5) response modulation. The purpose of the first two processes is to directly or indirectly change the environment that has induced the emotion (Gross, 2014; Martins et al., 2016). Attention deployment can be defined as the redirection of attention in a given situation to modify one’s emotions. Cognitive change involves the reappraisal of a situation to influence its emotional significance. The final process of response modulation consists in modifying experiential, behavioral or physiological components of the emotional response (e.g., by using relaxation, drugs, etc.) (Gross, 2014).

The cognitive emotion regulation questionnaire (CERQ) was designed to assess the type of cognitive emotion regulation strategies that an individual uses in response to an unpleasant event of daily life (Jermann et al., 2006). This measure assesses four maladaptive and five adaptive cognitive emotion regulation strategies. Self-blame refers to blaming oneself for when experiencing an unpleasant situation. Blaming others refers to holding others responsible when you experience an unpleasant situation. Rumination refers to thinking about the feelings and thoughts associated with unpleasant situations. Catastrophizing refers to having thoughts that emphasize the negativity of the situation. Putting into perspective refers to comparing the unpleasant situation to another situation. Positive refocusing refers to thinking about joyful and pleasant issues instead of thinking about the unpleasant situation. Positive reappraisal refers to thinking about the positive personal growth resulting from an unpleasant situation. Acceptance means accepting the reality of an unpleasant situation that is experienced. Finally, refocusing on planning refers to thinking about how to cope with an unpleasant situation. The CERQ is in line with the cognitive aspects of Gross’s model of emotion regulation (Martins et al., 2016). Indeed, the adaptive emotion regulation strategies assessed by the CERQ correspond either to attentional deployment (e.g., positive refocusing, refocusing on planning) or to cognitive change processes (e.g., positive reappraisal, putting into perspective) (Martins et al., 2016).

The relevance of the cognitive emotion regulation strategies assessed by CERQ was recently demonstrated in the domain of psychopathology (Martins et al., 2016; Potthoff et al., 2016). These authors observed an association between maladaptive cognitive emotion regulation strategies and symptoms of psychopathology (e.g., somatization, depression, and anxiety), while adaptive strategies seemed to be protective factors. Aldao and Nolen-Hoeksema (2010) even observed that the presence of maladaptive cognitive emotion regulation strategies had more damaging effects on psychological health than the relative lack of adaptive strategies. Response modulation consists mainly in inhibiting emotion expression, and has regularly been associated with negative affect and psychological distress (Lynch et al., 2001; Veilleux et al., 2014), and deficits in adaptive emotional regulation strategies (Veilleux et al., 2014). Individuals who lack proper adaptive emotion regulation strategies tend to use activities leading to immediate pleasure to alleviate negative emotions, but that may be harmful to the self and/or others. These activities may range from alcohol consumption (Baumeister et al., 2007; Merrill and Thomas, 2013; Veilleux et al., 2014), compulsive eating (Davis and Carter, 2009), unsafe sexual activities (Tice et al., 2001; Brawner et al., 2017), or cigarette smoking (Johnson and McLeish, 2016). In such instances, cognitive emotion regulation deficits lead to intense emotional distress from which the individual tries to obtain immediate relief, and also prevent him from making adaptive choices relevant for long-term personal goals (Tice et al., 2001). In other words, the unhealthy behaviors are a maladaptive emotional strategy motivated by the desire to escape the unpleasant awareness of one’s own emotional distress (Baumeister et al., 2007).

As a consequence of this tendency, it can be suggested that emotion regulation is tightly related to self-consciousness (SC), i.e., the persistent tendency of individuals to focus attention on the self (Baumeister et al., 2007; Fenigstein, 2009). Indeed, emotion regulation can indeed hardly take place without paying attention to the self (Scheier and Carver, 1977). Fenigstein et al. (1975) proposed a tridimensional construct of SC including private and public SC that refer, respectively, to the tendency to pay attention to internal aspects of oneself (e.g., thoughts, feelings, etc.) and the sensitivity to others’ opinion of oneself. Fenigstein et al. (1975) also added a third dimension, which is social anxiety. In his self-awareness model of alcohol consumption, Hull (1981) suggested that alcohol is frequently used as a means to reduce unpleasant awareness elicited by the experience of personal failure. In support of this theory, he observed that alcohol-dependent individuals (AD) scoring high in SC demonstrated a tendency to relapse rapidly after detoxification when they experienced situations of personal failure (Hull et al., 1983, 1986). Consistent with Hull’s theory, de Timary et al. (2013) observed that depression symptoms were strongly related to alcohol craving in ADs scoring high on SC, while no such relationship was observed in those with low SC scores. The role of self-related distress in highly self-conscious AD subjects was confirmed by the observation that self-discrepancy, i.e., the difference between the actual self and the ideal self (Higgins, 1987), was related to greater depressive symptoms, alcohol craving and alcohol consumption (Poncin et al., 2015), but also to less adaptive emotion regulation strategies, as measured by the CERQ. Self-consciousness moderated the relationship between the distress related to self-discrepancy and emotion regulation.

These observations support, for the AD population, Hull’s self-awareness theory of drinking (1981). However, Hull’s theory is not restricted to the AD population and it would be worth testing whether these dimensions also play a role in binge drinking, a milder form of excessive drinking, that is frequently observed at a younger age. Binge drinking is an alcohol consumption pattern defined by alternating episodes of intense alcohol intake and abstinence (Crego et al., 2009). According to National Institute of Alcohol Abuse and Alcoholism [NIAAA] (2004), a binge drinking episode is characterized by drinking four or more drinks for women and five or more drinks for men within a 2-h period. This alcohol pattern, which is widespread among undergraduate students, has damaging consequences such as cerebral and cognitive impact (Field et al., 2008). Binge drinking can also be considered as a risk factor for alcohol-dependence. Approximately 40% of AD individuals exhibit binge drinking habits during late adolescence (Bonomo et al., 2004; Jennison, 2004; Enoch, 2006). Furthermore, Maurage et al. (2012) proposed that binge drinking and alcohol-dependence were two stages of the same phenomenon, as they observed a similar pattern of cognitive impairment between binge drinkers (BD) and alcohol-dependent subjects. In the same vein, we believe it essential to investigate the relationship between emotional distress, SC, and emotion regulation in binge drinking, to identify whether BD also exhibit self-related sensitivity, as observed in the alcohol-dependent population.

Because, contrary to what is observed in the AD population, BD are not always exposed to ethanol consumption and do not exhibit persistent distress, we decided to investigate the relationship between cognitive emotion regulation strategies (using the CERQ), SC and emotional distress that was experimentally provoked in BD and control individuals. For this purpose, an anagram task where part of the task is unsolvable was chosen, because it allows an experience of failure and emotional distress to be induced (Miller, 2010). Whereas a lack of emotion regulation skills may lead individuals to experience greater emotional distress, we first postulated a positive relationship between the intensity of the emotional distress elicited by the anagram task and alterations in emotion regulation strategies in daily life, as measured by a self-rated questionnaire. If binge drinking may be considered as an unhealthy behavior to alleviate negative emotion because individuals lack skills to cope otherwise, we expected that the use of maladaptive emotion regulation strategies in daily life would be more related to distress in the anagram task among BD than in the control group. Finally, the third hypothesis was that SC moderates the relationship between emotional distress after failure in the anagram task and emotion regulation strategies in both groups.

## Materials and Methods

### Participants

A total of 3162 undergraduate students from the Université catholique de Louvain (Belgium) were screened with an online questionnaire assessing binge drinking habits. Among these students, 254 individuals meeting the criteria of control group and 246 individuals meeting recognized criteria characterizing moderate to intense binge drinking habits (Keller et al., 2007; Maurage et al., 2012) were recontacted by email. In order to participate in this research, students had to accept a fasting blood test that was conducted in the early morning to assess their inflammatory status but this issue is beyond the scope of this paper. Although students were financially incentivized to participate in the study, blood sample and early awakening were two reasons that have dampened their desire of participating in this study. Two groups of undergraduate students took part in this study (55 in total). The first group was composed of 32 students (19 men) having moderate to intense binge drinking habits that met the following criteria: (1) drinking 7 or more alcohol units per occasion, where a unit corresponds with 10 g of pure ethanol, (2) having 2 or more drinking occasion per week, (3) having a consumption speed of 3 or more units per hour. The second group consisted of 23 control individuals (9 men) who (1) drank fewer than 2 alcohol units per occasion, (2) had fewer than 0.5 drinking occasion per week, (3) drank less than 1 unit per hour, and (4) drank, on average, fewer than 2 units of alcohol per week. The average age was 20.88 (*SD* = 2.17) for BD and 21.78 (*SD* = 2.91) for the control group. None of the participants reported any personal or family history of substance dependence. No group difference was observed for age [*F*(1,53) = 1.763, *p* = 0.19, *d* = 0.36] nor gender [χ^2^(1, *N* = 55) = 2.195, *p* = 0.14]. This study protocol was approved by the Ethical Committee of the Hospital and the Medical Faculty of the Université catholique de Louvain. All subjects gave written informed consent in accordance with the Declaration of Helsinki.

### Measures

#### Procedure

Participants accomplished the anagram solution task (MacLeod et al., 2002; Watkins et al., 2008; Wemm et al., 2010) that consisted of 15 soluble and 15 insoluble anagrams, each five or six letters long. Each letter string from the anagram was displayed on a screen, in a random order, individually during 20 s. Then, a countdown of 10 s began and participants could type their answer. Feedback was given to the participant: “correct” for solved anagrams or “incorrect” for unsolved anagrams. Before starting, participants were instructed that on average 50–60% of anagrams were correctly solved and that their performance at this task would be a good indicator of future academic and career success. In other words, the instructor induced negative affect by providing a standard that students cannot reach. The anagram task was followed by a visual analog scale assessing distress experienced by participants (0 = not at all to 10 = extremely). After the anagram task, participants completed questionnaires assessing cognitive emotion regulation (CERQ) and SC (revised self-consciousness scale). On average, the experiment lasted about 25 min. At the end of the experiment, participants were debriefed about the goal of the anagram task, and no participant indicated that they were aware that it was impossible to reach the anagram standard given.

#### The Revised Self-Consciousness Scale (RSCS)

The self-consciousness trait was evaluated using Fenigstein et al.’s RSCS (Fenigstein et al., 1975; Scheier and Carver, 1985) that includes 22-items rated on a 4-point Likert scale (0 = extremely uncharacteristic to 3 = extremely characteristic). This measure is comprised of three subscales of *private self-consciousness* (i.e., attention to one’s inner feeling and thoughts), *public self-consciousness* (i.e., awareness of the self as a social object), *social anxiety* (i.e., discomfort in the presence of others). The items “I’m always trying to figure myself out,” “I care a lot about how I present myself to others,” and “It takes me time to get over my shyness in new situations” are some examples of private SC, public SC, and social anxiety, respectively. The internal reliability of the different subscales was acceptable to good, as shown by the Cronbach’s alphas: 0.69, 0.65, 0.82 for private SC, public SC, and social anxiety, respectively.

#### Cognitive Emotion Regulation Questionnaire (CERQ)

The objective of the CERQ is to evaluate how an individual generally copes with unpleasant situations. Thus, it measures cognitive aspects of emotion regulation and consists of 36-items, each of which is rated on a 5-point Likert scale ranging from 1 = almost never to 5 = almost always. This questionnaire comprises nine subscales: self-blame, blaming others, rumination, catastrophizing, putting into perspective, positive refocusing, positive reappraisal, acceptance and refocusing on planning. The first four subscales refer to maladaptive emotion regulation strategies, while the last five ones refer to more adaptive strategies (Garnefski et al., 2001; Jermann et al., 2006). The internal reliability of the different subscales was acceptable to excellent, as shown by the Cronbach’s alphas: 0.90, 0.70, 0.60, 0.83, 0.83 for self-blame, rumination, catastrophizing, blaming others and maladaptive strategies, respectively and 0.67, 0.82, 0.84, 0.86, 0.79, 0.90 for acceptance, positive refocusing, refocus on planning, positive reappraisal, putting into perspective and adaptive strategies, respectively.

### Statistical Analyses

Firstly, we conducted chi-squared tests and *t*-tests to compare groups on anagram distress, SC and emotion regulation strategies. We conducted regression analyses with anagram distress as the independent variable and emotion regulation strategies as the dependent variable. As the aim of this study was to determine whether binge drinking acts as a dichotomous moderator of the effect of anagram distress on emotion regulation strategies, a moderation analysis was employed. The PROCESS macro for SPSS developed by Hayes (2013) was used to examine moderation analyses. Dummy variables were created with ‘0’ representing the control group and ‘1’ representing the binge drinking group. The second interest of this study was to observe the influence of private and public SC on the relationship between anagram distress and emotion regulation strategies. We conducted moderation analyses using Hayes’ PROCESS macro. Private and public SC were considered to be the continuous moderators of the relationship between anagram distress and emotion regulation strategies. The Johnson–Neyman method allows determining the threshold values at which a moderator factor is responsible for a significant relationship between two variables (Hayes, 2013). Bootstrap confidence intervals were generated for regression coefficients in all tables. Considering that no options were available to calculate bootstrap inference for moderation analysis, we used Hayes’ hacking method to generate bootstrap confidence intervals (Hayes, 2013; Hayes, unpublished). It is worth mentioning that there were no missing data for all analyses.

## Results

### Description of Subject Population

There were no differences between binge drinking and control groups concerning the scores for anagram distress [*t*(53) = -0.520, *p* = 0.61, *d* = 0.14], public SC [*t*(53) = 0.109, *p* = 0.28, *d* = 0.30], private SC [*t*(53) = -0.91, *p* = 0.37, *d* = 0.24], self-blame [*t*(53) = 0.865, *p* = 0.40, *d* = 0.24], rumination [*t*(53) = 0.125, *p* = 0.90, *d* = 0.03], catastrophizing [*t*(53) = 0.846, *p* = 0.40, *d* = 0.24], Blaming others [*t*(53) = -0.044, *p* = 0.97, *d* = 0.01], maladaptive strategies [*t*(53) = 0.804, *p* = 0.43, *d* = 0.21], putting into perspective [*t*(53) = 0.08, *p* = 0.94, *d* = 0.02], positive refocusing [*t*(53) = 1.77, *p* = 0.08, *d* = 0.48], positive reappraisal [*t*(53) = 0.451, *p* = 0.65, *d* = 0.12], refocusing on planning [*t*(53) = 0.225, *p* = 0.82, *d* = 0.06] and adaptive strategies [*t*(53) = 1.288, *p* = 0.20, *d* = 0.21]. The control group used more acceptance strategies than the binge drinking group [*t*(53) = 2.557, *p* = 0.01, *d* = 0.69].

### Relationship between Anagram Induced Distress and Emotion Regulation across Subjects

Regression analysis revealed that anagram induced distress was significantly and positively related to blaming others only, β = 0.279, *t*(53) = 2.12, *p* = 0.04. The predictor explained 8% of the variance [*R*^2^ = 0.08, *F*(1,53) = 4.483, *p* = 0.04, *f*^2^ = 0.09]. In others words, the participants who exhibited higher distress when exposed to the anagram task were more likely to blame another person when they experienced an unpleasant situation. Conversely, the anagram induced distress was neither a predictor of maladaptive emotion regulation strategies [*R*^2^ = 0.05, *F*(1,53) = 2.731, *p* = 0.10, *f*^2^ = 0.05] nor of adaptive strategies [*R*^2^ = 0.00, *F*(1,53) = 0.023, *p* = 0.88, *f*^2^ = 0.00] across participants.

### Distress and Emotion Regulation in the BD and Control Groups

To examine the interactive effects of group variable and anagram distress on each emotion regulation strategy, moderation analyses were conducted using Hayes’ PROCESS macro. Analyses revealed that the effect of anagram distress variable on self-blame, rumination, and maladaptive emotion regulation strategies was different in the binge drinking and the control groups (**Tables 1**, **2**). In the BD group only, the anagram induced distress was related to more maladaptive emotion regulation strategies, rumination and self-blame.

**Table 1:** Multiple regression analyses predicting maladaptive emotion regulation strategies as a function of group (BD and C), distress and their interaction.

Model	β	*t*	*p*	*R*^2^	*f*^2^	*F*	*p*	Bootstrap CI (95%)
**Self-blame**				0.17	0.20	3.49	0.02	
Group	-1.09	-1.03	0.31					[-3.18; 0.96]
Distress	-0.16	-0.61	0.55					[-0.76; 0.51]
Distress × group	1.04	2.68	0.01	0.12	0.14	7.16	0.01	[0.21; 1.8]
*Condition effect of distress on Rumination for each group:*								
C (=0)	-0.16	-0.61	0.55					[-3.18; 0.96]
BD (=1)	0.88	3.04	0.003					[-2.15; 2.05]
**Rumination**				0.10	0.11	1.99	0.13	
Group	-0.17	-0.19	0.85					[-2; 1.58]
Distress	-0.18	-0.82	0.42					[-0.63; 0.29]
Distress × group	0.75	2.26	0.03	0.09	0.10	5.11	0.03	[-0.08; 1.40]
*Condition effect of distress on Rumination for each group:*								
C (=0)	-0.18	-0.82	0.42					[-2; 1.58]
BD ( = 1)	0.57	2.30	0.03					[-1.44; 2.4]
**Blaming others**				0.10	0.11	1.87	0.15	
Group	-0.01	-0.10	0.92					[-0.29; 0.23]
Distress	0.03	0.85	0.40					[-0.05; 0.08]
Distress × group	0.05	1.09	0.28	0.02	0.02	1.18	0.28	[-0.03; 0.14]
**Catastrophizing**				0.29	0.41	0.50	0.68	
Group	-0.12	-0.92	0.36					[-0.42; 0.16]
Distress	-0.01	-0.31	0.76					[-0.09; 0.09]
Distress × group	0.04	0.78	0.44	0.01	0.01	0.61	0.44	[-0.08; 0.14]
**Maladaptive strategies**				0.19	0.23	3.88	0.01	
Group	-2.27	-0.99	0.32					[-7.33; 2.33]
Distress	-0.26	-0.47	0.64					[-1.57; 1.18]
Distress × group	2.30	2.75	0.008	0.12	0.14	7.58	0.008	[0.54; 3.90]
*Condition effect of distress on Rumination for each group:*								
C (=0)	-0.26	-0.47	0.64					[-7.33; 2.33]
BD (=1)	2.04	3.27	0.002					[-5.11; 4.41]


*All β coefficients are unstandardized.*

**Table 2:** Multiple regression analyses predicting adaptive emotion regulation strategies as a function of group (BD and C), distress and their interaction.

Model	β	*t*	*p*	*R*^2^	*f*^2^	*F*	*p*	Bootstrap CI (95%)
**Acceptance**				0.13	0.15	2.50	0.07	
Group	-2.26	-2.48	0.02					[-3.93; -0.41]
Distress	-0.01	-0.05	0.96					[-0.5; 0.47]
Distress × group	-0.25	-0.75	0.46	0.009	0.009	0.56	0.46	[-1.03; 0.42]
**Positive refocusing**				0.06	0.06	1.14	0.34	
Group	-1.85	-1.78	0.08					[-3.90; 0.32]
Distress	0.10	-0.39	0.70					[-0.39; 0.65]
Distress × group	0.03	0.08	0.93	0.001	0.001	0.007	0.93	[-0.68; 0.67]
**Positive reappraisal**				0.05	0.05	0.97	0.41	
Group	-0.66	-0.53	0.60					[-3.08; 1.90]
Distress	0.49	0.30	0.11					[-0.30; 1.19]
Distress × group	-0.59	-1.31	0.20	0.03	0.03	1.72	0.20	[-1.63; 0.32]
**Putting into perspective**				0.009	0.009	0.15	0.93	
Group	-0.11	-0.11	0.92					[-2.25; 2.09]
Distress	0.16	0.61	0.55					[-0.65; 0.87]
Distress × group	-0.23	-0.59	0.56	0.007	0.007	0.35	0.56	[-1.13; 0.68]
**Refocus on planning**				0.008	0.008	0.15	0.93	
Group	-0.19	-0.18	0.85					[-2.11; 1.86]
Distress	-0.05	0.25	0.85					[-0.67; 0.61]
Distress × group	-0.12	-0.32	0.75	0.002	0.002	0.10	0.75	[-1.08; 0.71]
**Adaptive strategies**				0.04	0.04	0.78	0.51	
Group	-5.06	-1.29	0.20					[-13; 3.23]
Distress	0.68	0.72	0.47					[-1.74; 2.85]
distress × group	-1.16	-0.81	0.42	0.01	0.01	0.65	0.42	[-4.52; 1.77]


*All β coefficients are unstandardized.*

### Relationship between Anagram Elicited Distress, Emotion Regulation, and Private or Public Self-Consciousness in the BD and C Groups

To investigate the influence of private and public SC on the relationship between anagram distress and emotion regulation strategies, moderation analysis was conducted with Hayes’ PROCESS macro. The effect of anagram induced distress on blaming others and refocusing on planning strategies depended on private SC in BD (**Tables 3**, **4**). Compared to high self-conscious BD, low self-conscious BD who felt distress in response to the anagram task were more likely to blame others and refocus less on planning. Moreover, public SC moderated the relationship between anagram distress and acceptance: anagram distress was related to less acceptance among low self-conscious BD (**Tables 5**, **6**). No influence of private SC on the relation between anagram distress and emotion regulation strategies has been observed among the control group (**Tables 7**, **8**). Public SC moderated the relationship between anagram distress and adaptive emotion regulation strategies among the control group only (**Tables 9**, **10**). Anagram distress was related to more positive refocusing, positive reappraisal, refocusing on planning and adaptive strategies among high self-conscious participants in the control group. Conversely, anagram distress was related to less positive refocusing, refocusing on planning and adaptive strategies among low self-conscious control participants. **Table 11** shows the different correlations between all investigated variables.

**Table 3:** Multiple regression analyses predicting maladaptive emotion regulation strategies as a function of private SC, distress and their interaction in the binge drinking group.

Model	β	*t*	*p*	*R*^2^	*f*^2^	*F*	*p*	Bootstrap CI (95%)
**Self-blame**				0.40	0.67	6.15	0.002	
Private SC	0.25	1.46	0.16					[-0.08; 0.49]
Distress	0.80	3.25	0.003					[0.39; 1.37]
Distress × private SC	0.08	1.30	0.20	0.03	0.03	1.69	0.20	[-0.02; 0.19]
**Rumination**				0.25	0.33	3.07	0.04	
Private SC	0.17	0.99	0.33					[-0.28; 0.57]
Distress	0.51	2.11	0.04					[-0.27; 0.96]
Distress × private SC	0.07	1.22	0.23	0.04	0.04	1.49	0.23	[-0.07; 0.28]
**Blame to others**				0.31	0.45	4.29	0.01	
Private SC	0.003	0.13	0.90					[-0.03; 0.06]
Distress	0.08	2.73	0.01					[0.009; 0.14]
Distress × private SC	-0.02	-2.43	0.02	0.14	0.16	5.90	0.02	[-0.04; -0.002]
*Johnson–Neyman significance region*								
Value SC	% below		% above					
1.07	46.88		52.13					
**Catastrophizing**				0.30	0.42	4.07	0.02	
Private SC	0.05	3.06	0.005					[0.01; 0.08]
Distress	0.02	0.76	0.45					[-0.04; 0.07]
Distress × private SC	0.0009	0.15	0.88	0.0006	0.0006	0.02	0.88	[-0.01; 0.02]
**Maladaptive strategies**				0.50	1	9.18	0.0002	
Private SC	0.67	2.17	0.04					[-0.05; 1.30]
Distress	1.90	4.26	0.0002					[0.67; 2.62]
Distress × private SC	0.05	0.48	0.63	0.004	0.004	0.23	0.63	[-0.14; 0.38]


*All β coefficients are unstandardized.*

**Table 4:** Multiple regression analyses predicting adaptive emotion regulation strategies as a function of private SC, distress and their interaction in the binge drinking group.

Model	β	*t*	*p*	*R*^2^	*f*^2^	F	*p*	Bootstrap CI (95%)
**Acceptance**				0.14	0.16	1.47	0.24	
Private SC	-0.17	-1.06	0.30					[-0.61; 0.16]
Distress	-0.26	-1.12	0.27					[-1.06; 0.38]
Distress × private SC	0.10	1.70	0.10	0.09	0.10	2.89	0.10	[-0.10; 0.29]
**Positive refocusing**				0.02	0.02	0.23	0.87	
Private SC	-0.13	-0.66	0.52					[-0.74; 0.16]
Distress	0.14	0.51	0.61					[-0.57; 0.75]
Distress × private SC	0.03	0.40	0.69	0.006	0.006	0.16	0.69	[-0.10; 0.25]
**Positive reappraisal**				0.005	0.005	0.05	0.99	
Private SC	0.02	0.10	0.92					[-0.42; 0.46]
Distress	-0.11	-0.34	0.74					[-1.13; 0.46]
Distress × private SC	-0.01	-0.15	0.88	0.0008	0.0008	0.02	0.88	[-0.23; 0.17]
**Putting into perspective**				0.14	0.16	1.50	0.24	
Private SC	-0.33	-2.09	0.05					[-0.65; 0.01]
Distress	-0.03	-0.13	0.90					[-0.70; 0.45]
Distress × private SC	0.04	0.77	0.45	0.02	0.02	0.59	0.45	[-0.11; 0.17]
**Refocus on planning**				0.16	0.19	1.78	0.17	
Private SC	0.03	0.18	0.86					[-0.29; 0.42]
Distress	-0.22	-0.88	0.39					[-1.07; 0.22]
Distress × private SC	0.12	2.03	0.05	0.12	0.14	4.10	0.05	[-0.05; 0.24]
*Johnson–Neyman significance region*								
Value SC	% below		% above					
-4.55	9.38		90.63					
**Adaptive strategies**				0.06	0.06	0.60	0.62	
Private SC	-0.58	-0.84	0.41					[-2.35; 0.85]
Distress	-0.47	-0.48	0.64					[-4.01; 1.53]
Distress × private SC	0.28	1.15	0.26	0.04	0.04	1.32	0.26	[-0.40; 1.08]


*All β coefficients are unstandardized.*

**Table 5:** Multiple regression analyses predicting maladaptive emotion regulation strategies as a function of public SC, distress and their interaction in the binge drinking group.

Model	β	*t*	*p*	*R*^2^	*f*^2^	*F*	*p*	Bootstrap CI (95%)
**Self-blame**				0.31	0.45	4.17	0.01	
Public SC	0.13	0.96	0.35					[-0.13; 0.36]
Distress	0.89	3.34	0.002					[0.44; 1.45]
Distress × public SC	0.06	0.79	0.43	0.02	0.02	0.63	0.43	[-0.14; 0.19]
**Rumination**				0.30	0.43	4.17	0.01	
Public SC	0.30	2.51	0.02					[0.003; 0.55]
Distress	0.62	2.64	0.01					[-0.03; 1.03]
Distress × public SC	0.007	0.11	0.91	0.0003	0.0003	0.01	0.91	[-0.14; 0.18]
**Blaming others**				0.18	0.22	2.09	0.12	
Public SC	-0.008	-0.46	0.65					[-0.04; 0.04]
Distress	0.08	2.35	0.03					[-0.007; 0.13]
Distress × public SC	-0.008	-0.86	0.40	0.02	0.02	0.74	0.40	[-0.03; 0.02]
**Catastrophizing**				.07	.08	.70	.56	
Public SC	0.008	0.58	0.57					[-0.02; 0.04]
Distress	0.03	0.98	0.34					[-0.04; 0.09]
Distress × public SC	0.007	0.94	0.35	0.03	0.03	0.89	0.35	[-0.01; 0.03]
**Maladaptive strategies**				0.44	0.79	7.37	0.0009	
Public SC	0.41	1.71	0.10					[-0.07; 0.83]
Distress	2.09	4.46	0.0001					[0.10; 2.88]
Distress × public SC	0.05	0.40	0.69	0.003	0.003	0.16	0.69	[-0.21; 0.33]


*All β coefficients are unstandardized.*

**Table 6:** Multiple regression analyses predicting adaptive emotion regulation strategies as a function of public SC, distress and their interaction in the binge drinking group.

Model	β	*t*	*p*	*R*^2^	*f*^2^	*F*	*p*	Bootstrap CI (95%)
**Acceptance**				0.21	0.27	2.47	0.08	
Public SC	0.02	0.16	0.88					[-0.24; 0.28]
Distress	-0.30	-1.33	0.19					[-0.85; 0.17]
Distress × public SC	0.15	2.45	0.02	0.17	0.20	6.02	0.02	[-0.01; 0.28]
*Johnson–Neyman significance region*								
Value SC	% below		% above					
-1.34	50		50					
**Positive refocusing**				0.04	0.04	0.36	0.78	
Public SC	-0.05	-0.37	0.71					[-0.44; 0.34]
Distress	0.11	0.38	0.70					[-0.40; 0.67]
Distress × public SC	0.06	0.78	0.44	0.02	0.02	0.60	0.44	[-0.09; 0.25]
**Positive reappraisal**				0.05	0.05	0.53	0.66	
Public SC	-0.18	-1.15	0.26					[-0.52; 0.08]
Distress	-0.14	-0.46	0.65					[-0.93; 0.40]
Distress × public SC	0.02	0.20	0.84	0.001	0.001	0.04	0.84	[-0.18; 0.18]
**Putting into perspective**				0.01	0.01	0.09	0.96	
Public SC	-0.02	-0.18	0.86					[-0.26; 0.20]
Distress	-0.08	-0.34	0.73					[-0.64; 0.38]
Distress × public SC	0.02	0.37	0.72	0.005	0.005	0.14	0.72	[-0.09; 0.14]
**Refocus on planning**				0.16	0.19	1.81	0.17	
Public SC	-0.11	-0.90	0.37					[-0.37; 0.09]
Distress	-0.22	-0.89	0.38					[-0.88; 0.29]
Distress × public SC	0.12	1.87	0.07	0.10	0.11	3.48	0.07	[-0.03; 0.30]
**Adaptive strategies**				0.10	0.11	1.05	0.38	
Public SC	-0.35	-0.71	0.48					[-1.54; 0.70]
Distress	-0.63	-0.65	0.52					[-3.16; 1.17]
Distress × public SC	0.37	1.42	0.17	0.06	0.06	2.02	0.17	[-0.21; 0.98]


*All β coefficients are unstandardized.*

**Table 7:** Multiple regression analyses predicting maladaptive emotion regulation strategies as a function of private SC, distress and their interaction in the control group.

Model	β	*t*	*p*	*R*^2^	*f*^2^	*F*	*p*	Bootstrap CI (95%)
**Self-blame**				0.06	0.06	0.42	0.74	
Private SC	0.11	0.67	0.51					[-0.23; 0.47]
Distress	-0.16	-0.52	0.61					[-0.84; 0.62]
Distress × private SC	-0.03	-0.67	0.51	0.02	0.02	0.46	0.51	[-0.17; 0.09]
**Rumination**				0.29	0.41	2.56	0.09	
Private SC	0.28	2.49	0.02					[0.10; 0.59]
Distress	-0.16	-0.82	0.42					[-0.68; 0.32]
Distress × private SC	-0.02	-0.59	0.56	0.01	0.01	0.35	0.56	[-0.14; 0.06]
**Blaming others**				0.08	0.09	0.52	0.67	
Private SC	0.01	0.62	0.54					[-0.04; 0.07]
Distress	0.03	0.75	0.46					[-0.07; 0.09]
Distress × private SC	-0.004	-0.73	0.48	0.03	0.03	0.53	0.48	[-0.02; 0.006]
**Catastrophizing**				0.12	0.14	0.89	0.46	
Private SC	0.04	1.58	0.13					[-0.006; 0.09]
Distress	-0.008	-0.19	0.85					[-0.09; 0.11]
Distress × private SC	-0.002	-0.21	0.84	0.002	0.002	0.04	0.84	[-0.02; 0.02]
**Maladaptive strategies**				0.18	0.22	1.43	0.27	
Private SC	0.70	1.83	0.08					[-0.09; 1.85]
Distress	-0.23	-0.34	0.74					[-1.71; 1.41]
Distress × private SC	-0.08	-0.73	0.47	0.02	0.02	0.54	0.47	[-0.44; 0.20]


*All β coefficients are unstandardized.*

**Table 8:** Multiple regression analyses predicting adaptive emotion regulation strategies as a function of private SC, distress and their interaction in the control group.

Model	β	*t*	*p*	*R*^2^	*f*^2^	*F*	*p*	Bootstrap CI (95%)
**Acceptance**				0.01	0.01	0.07	0.97	
Private SC	-0.05	-0.35	0.73					[-0.31; 0.35]
Distress	-0.02	-0.08	0.94					[-0.61; 0.41]
Distress × private SC	-0.01	-0.33	0.74	0.006	0.006	0.11	0.74	[-0.14; 0.12]
**Positive refocusing**				0.03	0.03	0.16	0.92	
Private SC	-0.09	-0.60	0.56					[-0.52; 0.18]
Distress	0.09	0.32	0.75					[-0.49; 0.71]
Distress × private SC	-0.005	-0.11	0.92	0.0006	0.0006	0.01	0.92	[-0.13; 0.11]
**Positive reappraisal**				0.09	0.10	0.63	0.60	
Private SC	-0.05	-0.22	0.83					[-0.52; 0.35]
Distress	0.48	1.34	0.20					[-0.55; 1.13]
Distress × private SC	-0.007	-0.11	0.91	0.0006	0.0006	0.01	0.91	[-0.19; 0.18]
**Putting into perspective**				0.15	0.18	1.08	0.38	
Private SC	-0.18	-1.00	0.33					[-0.55; 0.19]
Distress	0.16	0.52	0.61					[-0.83; 0.77]
Distress × private SC	0.07	1.32	0.20	0.08	0.09	1.75	0.20	[-0.08; 0.22]
**Refocus on planning**				0.02	0.02	0.10	0.96	
Private SC	0.03	0.21	0.84					[-0.42; 0.29]
Distress	-0.05	-0.18	0.86					[-0.74; 0.70]
Distress × private SC	-0.02	-0.46	0.65	0.01	0.01	0.21	0.65	[-0.13; 0.13]
**Adaptive strategies**				0.03	0.03	0.23	0.88	
Private SC	-0.33	-0.52	0.61					[-1.78; 0.84]
Distress	0.66	0.60	0.56					[-2.45; 2.82]
Distress × private SC	0.02	0.11	0.91	0.0007	0.0007	0.01	0.91	[-0.44; 0.55]


*All β coefficients are unstandardized.*

**Table 9:** Multiple regression analyses predicting maladaptive emotion regulation strategies as a function of public SC, distress and their interaction in the control group.

Model	β	*t*	*p*	*R*^2^	*f*^2^	*F*	*p*	Bootstrap CI (95%)
**Self-blame**				0.05	0.05	0.34	0.80	
Public SC	-0.09	-0.43	0.67					[-0.52; 0.42]
Distress	-0.21	-0.66	0.52					[-0.80; 0.59]
Distress × public SC	0.07	0.80	0.44	0.03	0.03	0.63	0.44	[-0.12; 0.21]
**Rumination**				0.06	0.06	0.43	0.73	
Public SC	0.14	0.80	0.43					[-0.17; 0.54]
Distress	-0.21	-0.86	0.40					[-0.67; 0.31]
Distress × public SC	0.002	0.03	0.98	0.00	0.00	0.0006	0.98	[-0.22; 0.09]
**Blaming others**				0.11	0.12	0.82	0.50	
Public SC	-0.004	-0.14	0.89					[-0.05; 0.05]
Distress	0.04	1.11	0.28					[-0.05; 0.10]
Distress × public SC	-0.01	-1.33	0.20	0.08	0.09	1.77	0.20	[-0.03; 0.01]
**Catastrophizing**				0.02	0.02	0.13	0.94	
Public SC	0.02	0.57	0.58					[-0.04; 0.08]
Distress	-0.01	-0.31	0.76					[-0.12; 0.10]
Distress × public SC	0.0007	0.06	0.95	0.0002	0.0002	0.004	0.95	[-0.04; 0.02]
**Maladaptive strategies**				0.009	0.009	0.06	0.98	
Public SC	0.11	0.20	0.85					[-0.62; 1.10]
Distress	-0.29	-0.38	0.71					[-1.75; 1.46]
Distress × public SC	0.01	0.06	0.96	0.0002	0.0002	0.003	0.96	[-0.44; 0.25]


*All β coefficients are unstandardized.*

**Table 10:** Multiple regression analyses predicting adaptive emotion regulation strategies as a function of public SC, distress and their interaction in the control group.

Model	β	*t*	*p*	*R*^2^	*f*^2^	*F*	*p*	Bootstrap CI (95%)
**Acceptance**				0.14	0.16	1.07	0.38	
Public SC	-0.06	-0.34	0.74					[-0.31; 0.40]
Distress	-0.11	-0.48	0.64					[-0.62; 0.45]
Distress × public SC	0.11	1.79	0.09	0.14	0.16	3.2	0.09	[-0.06; 0.21]
**Positive refocusing**				0.38	0.61	3.86	0.03	
Public SC	-0.08	-0.49	0.63					[-0.42; 0.40]
Distress	-0.09	-0.41	0.69					[-0.50; 0.48]
Distress × public SC	0.21	3.37	0.003	0.37	0.59	11.37	0.003	[0.02; 0.37]
*Johnson–Neyman significance region*								
Value SC	% below		% above					
-2.78	21.74		78.26					
3.09	65.22		34.78					
**Positive reappraisal**				0.37	0.59	3.77	0.03	
Public SC	-0.38	-1.73	0.10					[-0.80; 0.01]
Distress	0.34	1.10	0.29					[-0.48; 1.02]
Distress × public SC	0.21	2.58	0.02	0.22	0.28	6.68	0.02	[0.06; 0.37]
*Johnson–Neyman significance region*								
Value SC	% below		% above					
1.38	52.17		47.83					
**Putting into perspective**				0.06	0.06	0.39	0.76	
Public SC	-0.14	-0.61	0.55					[-0.59; 0.48]
Distress	0.11	0.32	0.75					[-0.74; 0.99]
Distress × public SC	0.07	0.83	0.42	0.03	0.03	0.69	0.42	[-0.14; 0.26]
**Refocus on planning**				0.24	0.32	1.98	0.15	
Public SC	-0.06	-0.30	0.77					[-0.40; 0.42]
Distress	-0.21	-0.79	0.44					[0.87; 0.57]
Distress × public SC	0.17	2.43	0.03	0.24	0.32	5.88	0.03	[-0.02; 0.29]
*Johnson–Neyman significance region*								
Value SC	% below		% above					
-4.26	13.04		86.96					
8.38	95.65		4.35					
**Adaptive strategies**				0.36	0.56	3.60	0.03	
Public SC	-0.71	-1.08	0.29					[-1.93; 1.04]
Distress	0.03	0.04	0.97					[-2.29; 2.53]
Distress × public SC	0.77	3.12	0.006	0.33	0.49	9.75	0.006	[0.13; 1.23]
*Johnson–Neyman significance region*								
Value SC	% below		% above					
-4.36	13.04		86.96					
2.67	65.22		34.78					


*All β coefficients are unstandardized.*

**Table 11:** Correlations between all investigated variables.

	Anagram distress	Self-blame	Acceptance	Rumination	Positive refocusing	Refocusing on planning	Positive reappraisal	Putting into perspective	Catastro-phizing	Blame to others	Adaptive strategies	Maladaptive strategies	Public SC	Private SC
Anagram distress	1													
Self-blame	0.191	1												
Acceptance	-0.119	0.009	1											
Rumination	0.119	0.440***	0.041	1										
Positive refocusing	0.064	-0.109	0.337^∗^	-0.121	1									
Refocusing on planning	-0.077	-0.132	0.294^∗^	0.064	0.374^∗∗^	1								
Positive reappraisal	0.132	-0.263^†^	0.374^∗∗^	-0.035	0.375^∗∗^	0.665^∗∗∗^	1							
Putting into perspective	0.039	-0.354**	0.327^∗^	-0.233 ^†^	0.362^∗∗^	0.435^∗∗∗^	0.699^∗∗∗^	1						
Catastrophizing	0.029	0.504***	0.133	0.544^∗∗∗^	-0.046	-0.062	-0.298^∗^	-0.528^∗∗∗^	1					
Blame to others	0.279^∗^	0.073	-0.229 ^†^	0.065	-0.147	-0.118	-0.129	-0.076	-0.013	1				
Adaptive strategies	0.021	-0.239^†^	0.614^∗∗∗^	-0.079	0.660^∗∗∗^	0.754^∗∗∗^	0.865^∗∗∗^	0.776^∗∗∗^	-0.232	-0.186	1			
Maladaptive strategies	0.221	0.801***	0.001	0.766^∗∗∗^	-0.149	-0.093	-0.264	-0.431^∗∗∗^	0.744^∗∗∗^	0.359^∗∗^	-0.262	1		
Public SC	0.005	0.035	0.026	0.286^∗^	-0.020	-0.107	-0.200	-0.065	0.108	-0.070	-0.107	0.130	1	
Private SC	0.030	0.229	-0.127	0.377^∗∗^	-0.141	0.088	-0.042	-0.280^∗^	0.392^∗∗^	0.049	-0.134	0.378^∗∗^	0.141	1


*^∗^*p* < 0.05; ^∗∗^*p* < 0.01; ^∗∗∗^*p* < 0.001; ^†^0.05 < *p* < 0.10.*

## Discussion

The main objective of this study was to investigate the relationship between emotional distress, emotional regulation and SC in binge drinking. Individuals scoring high on SC and exhibiting poor emotion regulation skills are likely to experience unpleasant awareness of their emotional distress, and to thus use activities to relieve negative emotion. In an alcohol-dependent population, Hull et al. (1986) has already observed that subjects with high in SC used alcohol as a means of reducing awareness of personal failure and are more likely to relapse when they experience such failure. According to the continuum hypothesis suggesting that binge drinking and alcohol-dependence are two stages of the same process (Enoch, 2006; Maurage et al., 2012), it is important to explore in the BD population the potential Self-sensitivity for which activities leading to immediate pleasure (e.g., alcohol consumption) may be used as a means to relieve self-related emotional distress.

It is important to note that there were no differences between the BD and control groups regarding the overall scores of distress on the anagram task, SC and any cognitive emotion regulation strategies, except for acceptance, which was higher in the control group. The question that is raised by this study is, therefore, whether emotional regulation strategies and SC do relate in a specific way to experimentally induced emotional distress, and whether such a relationship might be specific to the BD group. We also examined the potential moderating impact of SC on the relationship between emotional distress and emotion regulation strategies.

The first objective of this study was to examine the relationship between emotional distress associated with the anagram task and emotion regulation strategies. As mentioned above, the anagram task concerned preoccupation with academic achievement (Miller, 2010), which is a central preoccupation in students. It is therefore an appropriate situation for evaluating sensitivity to self-threatening situations. In the overall group, anagram distress was positively related blaming others in unpleasant situations that participants experienced. Besides this relationship, we failed to observe any other relationship between emotional distress and emotion regulation strategies in the overall group. Duval and Silvia (2002) suggest that individuals who note a discrepancy between themselves and an ideal standard and who are unable to improve themselves, have the tendency to attribute their failure to external sources: this seems to be the case for a substantial proportion of the subjects irrespective of their drinking habits.

However, this does not rule out the existence of specific relationships between emotional distress and emotion regulation strategies in the BD or control groups and of a possible influence of SC, which were the subject of our second and third objectives, respectively. The main differences that we observed between the two groups in our data may be summarized in the following manner: (1) In the binge drinking population, greater distress induced by the anagram task was related to more self-blame, to more rumination and to more maladaptive emotion regulation strategies. Hence, binge drinking individuals who experience more distress when exposed to difficult situations are also those that describe a higher tendency for maladaptive strategies, and in particular, strategies that are related to a negative self-perception. This is an indirect observation that supports the role of self-related elements in binge drinking. (2) In control participants with higher levels of SC, greater distress induced by the anagram task was related to more adaptive strategies, and in particular, by more positive refocusing, more positive reappraisal and more refocusing on planning. Such a relationship was not observed in the binge drinking population. A possible explanation for this observation is that in non-binge drinking participants, higher levels of public SC are related to more active modes of coping, such as positive refocusing and reappraisal or refocusing on planning when exposed to negative events.

Overall, these observations support different modes of coping in response to a self-threatening situation among BD and non-binge-drinkers. Silvia and Duval (2001) suggested that different attitudes may be observed when a subject is exposed to situations where he/she does not measure up to their target standards: their first impulse may be to change their actions and attitudes in an attempt to measure up to the expected standards. We believe that this might be the case for the non-binge-drinking individuals that are high in public SC. SC hence appears to be a disposition that motivates them to improve actions and attitudes to meet self-standards. Conversely, some individuals may be overwhelmed by the self-discrepancy induced by the situation of failure, which may lead to maladaptive emotion regulation strategies, such as self-blame or rumination. This trend was observed in the binge drinking population. Considering that self-blame and rumination are regarded as the most self-contained cognitive strategies (Bornas et al., 2013), the observation that BD who express distress related to the self are also more likely to pay attention to the self and to use maladaptive, self-contained emotion regulation strategies in keeping with self-sensitivity in BD. Moreover, rumination and self-blame describe the tendency to focus on the causes, meanings and consequences of distressing situations and to attribute the causality of these situations internally, respectively, which in turn exacerbates psychological distress (Jermann et al., 2006; Moberly and Watkins, 2008). This could highlight a sensitivity to self-stressors in some BD. These results are in line with the observation that ADs are more likely to drink or relapse if they are more self-conscious (Hull et al., 1986; de Timary et al., 2013), and extend Hull’s self-awareness theory of alcohol-drinking to part of the BD population (Hull, 1981). These data are also consistent with Poncin et al.’s (2015) results suggesting that the sensitivity toward self-discrepancies is related to less adaptive emotion regulation strategies in the AD population. These results are suggestive of the importance of the sensitivity to self-stressors of a subgroup of BD, but does not allow us to ascertain whether this leads to alcohol consumption. However, Lannoy et al. (2017), suggest the existence of several binge drinking profiles, including an emotional profile for which alcohol is used as a maladaptive regulation strategy. This subgroup of BD could be more sensitive to stressful situations related to the Self and could use alcohol to relieve emotional distress.

Two other differences were also observed between BD and controls. BD, who had below average levels of private SC and who experienced distress from the anagram task, were less likely to refocus on planning and more likely to blame others. BD with low public SC and who experienced anagram distress used fewer acceptance strategies. These two observations suggest that low private/public SC in the binge drinking group may also be related to more maladaptive or less adaptive emotion regulation strategies. Decreasing self-consciousness might be a maladaptive way to escape self-stressors in some BD individuals.

This study is among the first to examine the relationship between self-regulation and SC in binge drinking. A limitation of this study was the small sample size. Field (2007) indicates that the sample size also depends on the effect size. For a regression with three predictors (as in this study), he recommends having a sample size of 40 for a large effect size. If the effect size is medium and small, the sample size should be 80 and 600, respectively. Considering the 3 parameters and the small sample size, regression models might be overfitting. It is thus important to be cautious about the results of this study that may not reflect the overall population. It is therefore necessary to repeat this study in a larger sample in order to increase statistical power and to be even more representative of alcohol consumption in student population. Moreover, to increase the variability of students’ consumption behaviors, it may be important to consider alcohol consumption habits as a continuous variable. Townshend and Duka (2002) propose a scoring method based on an alcohol use questionnaire in binge drinking. This score is calculated based on the number of drinks per week, the speed of drinking, number of times one was drunk in the previous 6 months and percentage of time being drunk when drinking. Moreover, another limitation of this study is that there was no manipulation check of emotional distress. Therefore, further studies should pay attention to check participants’ emotional state before the task inducing emotional distress. Finally, it could be interesting to distinguish investigate the effect of gender in further studies because there may be gender differences in emotion regulation strategies.

## Conclusion

This study supports the hypothesis of a difference in the relationship between self-regulation and emotional distress among BD and non-BD. Emotional distress was related to more self-blame, rumination and maladaptive regulation strategies in BD only. A sensitivity to self-stressors with difficulties of emotion regulation was also observed in BD. Moreover, this study suggests that public SC may motivate individuals to improve actions and attitudes to meet target standards among non-binge-drinkers. It is important to continue the careful exploration of the self-related elements model of alcohol consumption in alcohol-dependence and binge drinking, as the identification of shared self-related sensitivity in binge drinking and alcohol-dependence may inform preventative interventions.

## Author Contributions

All authors developed the study design. MP contributed to the data collection, performed the statistical analysis and wrote the original paper. PdT and NV oversaw the writing of the paper. All authors contributed to and have approved the final version of the manuscript.

## Conflict of Interest Statement

The authors declare that the research was conducted in the absence of any commercial or financial relationships that could be construed as a potential conflict of interest. The reviewer ER and handling Editor declared their shared affiliation.
